# Medical Students’ Experience of Mindfulness Training in the UK: Well-Being, Coping Reserve, and Professional Development

**DOI:** 10.1155/2019/4021729

**Published:** 2019-02-03

**Authors:** Alice Malpass, Kate Binnie, Lauren Robson

**Affiliations:** 1 Centre for Academic Primary Care (CAPC), Population Health Sciences, Bristol Medical School, University of Bristol, Canynge Hall 39 Whatley Road, Bristol BS8 2PS, UK; 2 Pennine GP Training Scheme, West Yorkshire, UK

## Abstract

Medical school can be a stressful experience for students, resulting in stress-related mental health problems. Policy recommendations from the General Medical Council (GMC), the body responsible for improving medical education in the UK, recommend the use of mindfulness training to increase well-being and resilience to stress. Students participating in an eight-week mindfulness training between Autumn 2011 and Spring 2015 were invited to complete a free text survey at the end of their mindfulness course. In addition, six qualitative interviews were conducted lasting between 60 and 90 minutes. Interviews used a topic guide and were recorded and transcribed verbatim. We used the framework approach to analyse the data. Students reported a new relationship to their thoughts and feelings which gave a greater sense of control and resiliency, an ability to manage their workload better, and more acceptance of their limitations as learners. The small group context was important. Students described improved empathy and communication skills through building inner awareness of thoughts and feelings, noticing judgments, and developing attentive observation. The findings show how resiliency and coping reserve can be developed within medical education and the role of mindfulness in this process. We present a conceptual model of a learnt cycle of specific vulnerability and describe how MBCT intercepts at various junctures in this self-reinforcing cycle through the development of new coping strategies that embrace an “allowed vulnerability.”

## 1 Introduction

### 1.1 Stress and Mental Well-Being amongst University Students

Students across disciplines have higher levels of anxiety and low mood compared to the general population [1]. There is some evidence that medical students may be particularly at risk of developing mental health issues because medicine attracts psychologically vulnerable personalities before selection to medical school [2]. Some even argue that a proportion of medical students may be motivated to study medicine by unconscious neurotic drives and unresolved conflicts dating from their childhood in an attempt to improve their well-being by healing others [3–5]. Medical training itself accentuates this vulnerability by creating a learning culture which inhibits help-seeking. For these reasons, medical school can be a particularly stressful experience for students [6], resulting in stress-related mental health problems [7]. Medical students display high levels of depression and anxiety [8], with mean anxiety scores and standard deviation above those of general population and their depression scores increasing significantly throughout the first year of medical school [9]. Research shows that mental health worsens after students begin medical school and remains poor throughout training [5]. Research in the UK has consistently shown that stress and burnout are endemic during medical training [10, 11]. There has been debate about whether students develop mental health problems as a result of the hidden curriculum [12–14] or because they are more likely to have psychologically vulnerable personalities before selection to medical school, such as perfectionism [2–4].

There is increasing emphasis on emotional well-being in the educational policy in the UK. Policy recommendations from the General Medical Council (GMC), the body responsible for improving medical education in the UK by setting standards for students and doctors, recommend the use of mindfulness training to increase well-being and resilience to stress [15]. The internal GMC review on suicides amongst doctors states that “emotional resilience training should become an integral part of the medical curriculum” [16]. In Australia, mindfulness training is now incorporated into the medical curriculum [17], whereas in the UK, mindfulness for medical students is still in development.

### 1.2 Evidence Base of Mindfulness in Medical Student Populations

The integration of mindfulness in medical training has been related to fostering reflective practice and professionalism [18–20] as well improving psychological well-being and preventing burnout [17, 21]. Most of the trial evidence on the efficacy of mindfulness approaches in reducing stress, anxiety, and mood disturbance for medical students is based outside the UK, in the USA [22, 23] and Australia [24]. Despite this clinical evidence base, there has been no qualitative work exploring students’ experiences of mindfulness training in the countries where these trials took place. In the UK, there has there been no exploration of how mindfulness training is being implemented and with what impact, despite the recent GMC recommendations for mindfulness to form one strategy in supporting students’ mental health. This echoes a wider gap in the medical education literature which suggests “although much is known about student distress, little is known about student well-being, how it can be fostered, and its potential to enhance learning and professional development” (p. 1619) [5].

Our overall aim in this study was to explore UK medical students’ experience of taking part in a 8-week course of mindfulness training delivered by a qualified teacher who adhered to the Good Practice Guidelines for Mindfulness Teachers [25]. The course did not form part of the core medical curriculum. Course content followed the manualised curriculum of “Mindfulness-Based Cognitive Therapy” [26] and required students to attend for two hours each week and commit to 30-minute daily home practice in between sessions. For a description of thematic content, see Table 1. Instruction consists of various formal and informal meditation practices, including guided body scans, sitting and walking meditations, mindful movement (based on Hatha yoga), 3-minute breathing spaces, and focused awareness on routine daily activities [27]. Our objective was to evaluate through qualitative methods the impact of attending an eight-week course upon emotional well-being and students’ ability to manage workload and stressful situations. We were also interested whether students reported any impact of mindfulness training on professional development, such as communication skills or empathy.

## 2 Methods

### 2.1 Recruitment

Mindfulness training took place within one medical faculty in the southwest of the UK. 57 students were referred to a mindfulness group either by their GP or student advisor between 2011 and 2015. For recruitment to the qualitative interviews, students gave verbal consent to their mindfulness teacher to be contacted by a researcher after the mindfulness course had finished. LR invited students to take part in an interview by e-mail, and students gave written consent for their data to be analysed before the interview was conducted. 12 students were approached for an interview and six students agreed (Table 2). In addition to the qualitative interviews, all 57 students who had participated in mindfulness training between Autumn 2011 and Spring 2015 were invited to complete a short survey with open-ended text responses, at the end of their course.

### 2.2 Data Collection

LR conducted all the interviews face-to-face which lasted between 60 and 90 minutes. LR was a final year medical student and so had “insider” knowledge on the medical school culture and curriculum in which the participants were located. Insider knowledge is discussed in the literature as both a potential strength and weakness [28]. Engagement of nonprofessional researchers in data collection is seen as both a means of “minimising harm in the research process” (p. 33) [29] and a “moral dilemma” (p. 598) [30] when closeness increases the risk of exploitation [31]. Trustworthiness was established between LR and interviewees through the latter being introduced to LR via e-mail by their MBCT teacher whom they trusted. LR also gained trust from interviewees by disclosing her “insider” status as a final year medical student who would empathise with help-seeking for mental health issues. We took the view that the “insider status” of LR was a strength and would encourage an open and honest exploration amongst interviewees. We reflect further on this under limitations at the end of the paper.

Interviews used a topic guide (Table 3) and were recorded and transcribed verbatim. The questions in the survey are listed in Table 4.

### 2.3 Data Analysis

We used the framework approach [32] to analyse the data as this suited the qualitative and free text survey data we had gathered. Framework analysis involves placing the interview or survey questions as column headings and entering each participant in a separate row. Verbatim data were put in the corresponding cell. The framework approach allows for thematic analysis, allowing emerging themes (that stray outside the original topic guide or survey question) to be entered as additional column headings. Comparisons can be made between participants and across themes without losing the continuity of each participant’s experience. The authors felt a saturation of key themes had been reached with six in-depth interviews in combination with analysis of the free text survey data from 57 students. The methodological approach in forming our study design and choice of approach to data collection and analysis views interviewees as active contributors to the knowledge that is generated through interviews (as opposed to viewing interviewees as subjects being studied). Our methodological position views the data created in the interviews as a process of coproduction between LR and interviewees and the analytic process as iterative and interpretative.

## 3 Results

The survey data show that, of the 57 students who attended a mindfulness course, 15 were male and over half the students attending an 8-week course were from years 2 and 3 although students from all year groups attended mindfulness training (Table 5). The findings from the qualitative interviews and open-ended survey questions show students described personality traits described in the literature that predispose medical students to developing mental health issues. Students reported a learnt awareness of stress triggers and early warning signs of stress symptoms. Students reported a new relationship to their thoughts and feelings which gave a greater sense of control and resiliency. Students described the importance of the mindfulness training to go beyond learning a set of tools for coping with difficulty. They described a complete change in attitude: a “new way of looking at life” (Yr4, S2), and a complete change in perspective: “its changed the way I experience the world and my own emotions. Set me on a path towards healthy processes” (Yr2, S3). The small group context was an important facilitator for these changes in well-being, resiliency, and outlook. Students also reflected on the impact of mindfulness training in their development as clinicians. The findings explore these themes with verbatim examples from the qualitative data and free text survey data.

### 3.1 Motivations for Attending an 8-Week Course

Our findings on motivations for attending a mindfulness course illustrate some of the individual and systemic factors of stress discussed in the literature that may predispose medical students more to developing mental health issues than their non-medical student peers. For example, interviewees described perfectionism, being a high achiever and the impact of a competitive environment:

I think all medics are really driven, they set high expectations and try and do a lot of things so maybe we get more overwhelmed because there’s so much to do. I think you are used to being the best, I am the clever person that is my identity … I’m not the funny one, the loud one, the party one, I’m the clever one and then you come into Uni and you’re not the clever one anymore because everyone’s clever and everything’s hard and you can’t keep up with it all so you completely lose who you are and at the same time there’s no-one to help you. (E, 1st year, C1)

For one student, an existing maladaptive coping strategy was intensified by the context of studying medicine, which had led her to suspend her studies after a period in hospital for an eating disorder:

I mean I say I was fine before uni, it depends how you look at an eating disorder, like if you look at it in terms of food and exercise, I was fine before uni, my favourite food was pork pie sandwiches. But in terms of work I was not fine before uni, I’ve always been, a perfectionist, “I need to work really, really hard and I need to get high grades” and I guess at uni that just exploded a bit more and just came out through eating [disorder] I think. So in terms of my work life balance I never really had that right but at uni its almost like I just latched onto food as a way of managing I suppose. (R, 2nd year, C4)

This student took the mindfulness course during her year of suspended study to support her return to medicine.

### 3.2 Awareness of Stressors and Early Warning Signs

The 8-week course teaches students to recognise their unique early warning signs that indicate they may be slipping into maladaptive coping strategies in response to stress:

I now notice more when I’m getting stressed and try and do something about it rather than just letting it be there and I am better at knowing what kind of things stress me out. (Yr3, IJ)

Being able to self-monitor their stress levels enabled students to prevent feeling overwhelmed:

Recognizing triggering events and stress cycles has really helped prevent “melt downs.” (Yr2, S3)

This self-knowledge provided students with a sense of control:

I feel more in control now, like I have a resource to fall back on, something to help me. (Yr2, S30)

This feeling for a fifth-year student left her “more resilient to difficulties” (Yr5, S22) and for a first-year student she felt she had “new and better ways of coping” (Yr1, S25).

### 3.3 New Relationship to Thoughts

Identifying early warning signs could be achieved through noticing thoughts and having a new relationship to them:

I realised I was always exploding everything into a global thought, creating a big story and stressing myself out. … the course has changed my mindset, how I think of myself, how I react to situations, before I would be sitting doing some work, thinking “oh my god this is so hard, I don’t understand it, I’m not going to do well, I’m going to fail my exams.” [Now] as soon as those thoughts start, I recognise them, and can cut them off, instead of wasting my time stressing, I just think, “look, its not going to happen (failure),” I think “ok, what can I do differently”? if I don’t understand, get another text book, I recognise when I’m starting on that downward spiral [of negative thoughts] … (Yr1, IN)

Realising “thoughts are not facts” enabled students to take a step back from negative thought patterns and respond in new ways:

If I don’t get work done I don’t beat myself up about it. Mindfulness teaches me to be kinder to myself. (Yr2, S4)

Students recognised that their negative thoughts were related to habits of striving for perfection that no longer served them:

I was very judgemental with myself and was striving towards an idea of perfection that I couldn’t achieve. I’ve noticed I am a lot more accepting of my imperfections … I’ve learnt I need to be kinder to myself. (Yr2, S31)

Many students realised how their thoughts were unhelpful, even “manipulative” (Yr1, S26):

I understand that you can have separation from your thoughts, they aren’t certainties, they don’t run your life and maybe they aren’t always helping you. (Yr2, S24)

### 3.4 Concentration and Managing Workload

As a result of a new relationship to negative thoughts, students managed their workload differently, giving them a greater sense of efficiency:

I just recognise when I’ve got to that point where I’m not going to take anything else in, where I just need some time out to go away and come back … in terms of workload it’s just helped me be more efficient in my studies. (Yr1, IN)

Mindfulness techniques helped students unwind after working all day as well as refresh and regain concentration during the day:

I was using the three minute breathing space, at night it felt quite invaluable for helping me get to sleep, helping me to detach myself from my work and wind down a bit.…. During the day it was quite a useful tool to refresh myself, allow myself to kind of regain concentration and get back to work. (Yr2, IP)

Mindfulness helped students achieve a sense of balance and flexibility in relation to work:

I was very scared about changing how I worked, [mindfulness] gave me the flexibility to try something different, so I did stuff like not working in the evenings, and not working in the mornings [before lectures] and I found that (a) I was happier, (b) I did better academically and (c) I had time to do other stuff with my life … the neuro exam was the first test of doing things differently and that was the best exam result I’ve had in medicine, I worked hard for it but a realistic level of hard. (Yr3, IJ)

### 3.5 Acceptance (of Limitations)

Underlying students’ experience of having greater balance and flexibility in managing their workload was a shift in attitude, to give themselves permission to find things hard and for this not to be interpreted as a sign of failure:

Well I think accepting that things are difficult. You can’t do everything … everyone finds things difficult and it’s okay to find it difficult. So I think I took that [judgement] away. (Yr 2, IB)

Coupled with this sense of acceptance was a distancing from the competitive and comparative culture of medicine: “no-one needs to be compared to” (Yr2, S3) because “everybody is different and deals with things in a different way” (Yr3, S27).

### 3.6 The Small Group Learning Environment

A facilitator of the personal and work-related impacts of mindfulness is the small group learning environment. Despite at the beginning of an 8-week course students feeling “worried and nervous about sharing in a group” (Yr2, S3), nearly all students commented on the group as an “accepting, warm” (Yr2, S9), “welcoming, intimate and safe space” (Yr2, S4) in which they “bonded and developed” (Yr2, S10) with their peers. This process was described as “very empowering” (Yr2, S3) and “constructive” (Yr2, S6). The group space offered a source of hope:

I felt I came to a safe place each week where I could learn to be ok so it gave me hope. (Yr3, S15)

Students found listening to each other helpful. The group space normalised automatic negative thought patterns which helped students begin to view their thoughts differently:

The group discussions were helpful it was comforting to know other people had very similar thought patterns. (Yr1, S27)

Students found they are not alone in finding their course difficult, allowing them to let go of self-judgments that they are “not good enough”:

It helped me learn, I’m not the only one in my situation, at the beginning I thought I might be the most stressed person, over time everyone started opening up, everyone seemed quite receptive, open to each other, quite supportive. (Yr2, IE)

### 3.7 Self-Care and Self-Support

The result of a greater sense of self-acceptance is that students begin to spend more time improving their social support networks:

When I’m stressed I have a tendency to withdraw and not socialise but now I invest in relationships … when I started medical school I cut off from everyone, saying “I don’t have time, I just need to work,” I had to make the conscious effort of pushing myself, saying, “building relationships is important, it’s part of self care and having people supporting you.” Now, when I’m socialising with people, instead of sitting there and thinking, “oh, I could be at home doing work,” I am now saying, “you’re looking after yourself, time out is good,” allowing me to enjoy that instead of feeling guilty about it. (Yr1, IN)

The mindfulness course invites students to consider the balance of nourishing and depleting activities in their lives and to make some changes. For some students, this works as an important encouragement and permission to self-care:

The course made me think about how to look after myself better what’s good about Mindfulness is that it’s someone telling you to look after yourself and I think that’s quite important. (Yr2, IE)

### 3.8 Empathy

For some students, mindfulness had helped them cultivate empathy through noticing their reactions towards patients:

I’m mindful of my own reaction towards patients and their stories and the hospital situation and thinking about patient’s conditions and their suffering … I’d like to say that it’s improved my ability to empathise with patients and what’s going on with them. (Yr2, IP)

The ability to relate to what patients are saying is connected to students being aware of their own thought patterns:

I think mindfulness has helped make me more empathetic, I wasn’t ever nasty before, I was very sympathetic, but I probably couldn’t actually relate to what somebody was saying as much, whereas now I notice more, I think becoming more aware of how I think about things has made me more aware of how other people might think about things, I guess that would make me a better practitioner. (Yr3, IJ)

The key here is the link the student has made between developing inner awareness and patient care.

### 3.9 Less Judgemental of Patients

Mindfulness training had helped students notice and step back from “judging thoughts.” This translated into clinical contexts as noticing when other doctors were judging patients:

I hear a lot of doctors say things like, “oh you know one of those typical middle aged, overweight, chronic fatigue type patients” and now when I hear someone say that I’m “ok well you might have that sort of judgement about that person” but that person is still living with an illness that’s affecting them, so that’s probably the important bit right now, it’s just made me a bit more aware. (Yr3, IJ)

The link in our data between empathy and awareness of judging thoughts resonates with work that theorises the link between mindfulness and empathy: “to be empathic I must witness and understand the patient’s suffering and my [or others] reactions to the patients suffering” (p. 836) [17].

### 3.10 Communication Skills

Students valued mindfulness communication styles, seeing their relevance for clinical practice:

Through observing how the sessions were run but also through personal practice. Its not necessarily something that would be acknowledged as a communication skill under the traditional framework but [mindfulness leads to] feeling more confident in talking about feelings the approach of the [mindfulness] sessions was an open, friendly, exploration of thoughts and feelings and maybe that’s helped me give patients the space to express themselves. (Yr2, IP)

Mindfulness training teaches students to be curious about their own feelings and thoughts, and this then grows into a curiosity about the patient’s feelings and thoughts. Epstein suggests “curiosity is central both to caring about the patient as well as solving problems” (p. 836) [17].

## 4 Challenges to Engaging with Mindfulness

### 4.1 The Body

Throughout an 8-week mindfulness course, students are invited to inhabit their body and are asked to locate thoughts, feelings, and impulses as sensations in the body. For medical students, invitations to come into contact with the body were sometimes met with difficulty, as for many of them “medical neutrality had spilt over into their personal lives” (p. 65) [33], especially if they arrived to a session having just left an anatomy class involving dissection. For example, a focus on the breath was often challenging as medical anatomic theory would intrude, especially during invitations to breath into the edges of pain or discomfort, leading students to “think about the breath” instead of having a direct experience of the breath in terms of sensation and movement.

### 4.2 Thoughts

Although students found the theme “thoughts are not facts,” one of the most helpful themes on the course, noticing self-critical thoughts and relating differently to thoughts were often challenging and described as an obstacle. Some experienced a “reluctance to let negative thoughts and feelings in” (Yr2, S3), whilst others felt they were “battling” (Yr2, S4) with their thoughts. For some students, the course was the first time they saw clearly their self-judgemental thought patterns and felt their biggest obstacle was “their own mind, berating myself” (Yr3, S28), from which they wanted to “run away” (Yr3, S18) to avoid feeling “overwhelmed” (Yr2, S33). Most students overtime were able to work through this obstacle:

I found alot of the course very challenging as it brought up and amplified a lot of thoughts that were very difficult for me to deal with. However it has helped me to look at them differently and although its still very hard I have noticed changes and improvements. (Yr1, S25)

## 5 A Model of How Mindfulness Breaks a Learnt Cycle of Vulnerability

**Figure 1:**
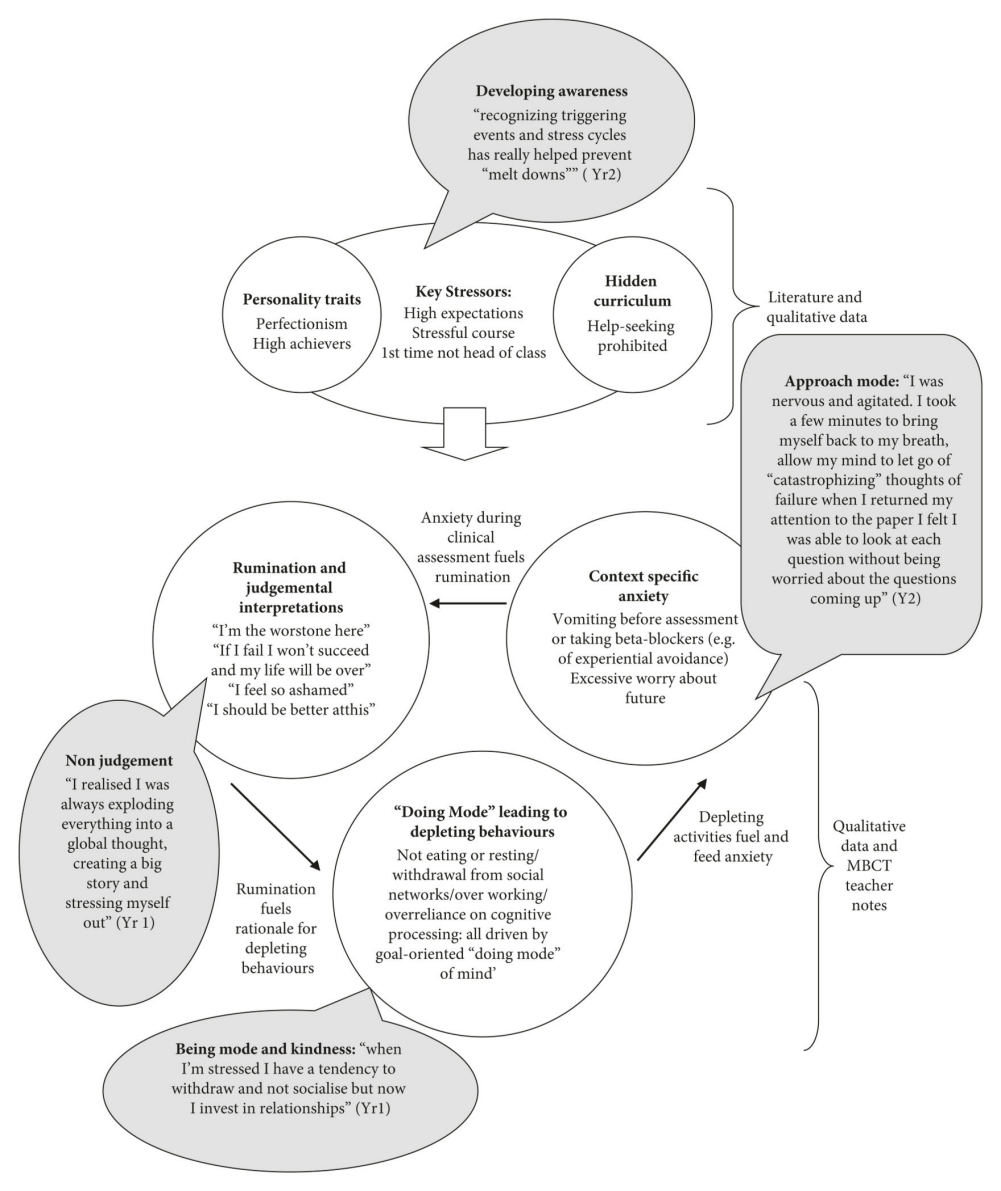
An initial model of learnt vulnerability amongst medical students: how MBCT may help.

Figure 1 shows how we have mapped the learnt cycle of vulnerability based on our reading of the literature on what predisposes medical students to developing mental health issues, combined with our analysis of qualitative data and MBCT teaching case notes. Figure 1 also shows how mindfulness may intercept this learnt cycle of vulnerability. In Figure 1, we can see that medical students have a strong tendency to worry about what might happen in exams and clinical assessments, often avoiding being with their direct experience of anxiety in the body with beta-blockers or until they vomit whilst on the ward. This context specific anxiety fuels ruminative-type thoughts about being a failure and not good enough. Harsh self-judgement and not being able to cope then feed a sense of needing to try harder with longer working hours. Nourishing activities (exercising, eating, and seeing friends) are stopped. The lack of nourishing activities fuels the context-specific anxiety of medical training. In our working model, medical students find themselves in a vicious, self-reinforcing cycle of stress. Coping strategies, such as working harder, which may have been successful in past episodes of stress, perpetuate the cycle, reinforcing negative, ruminative thinking about “failing to cope” and “being the worst student on the course.” Underlying this viscious cycle are two operandi: the “doing mode” of mind which is goal-focused and the influence of the hidden curriculum upon creating a clinical culture of invulnerability which leads, for example, to the avoidance of help-seeking. The benefits of mindfulness training we have reported in our findings are presented in Figure 1 as verbatim exemplars of how what is learnt during the MBCT course intercepts this learnt cycle of vulnerability, for example, developing awareness of stressors and automatic habitual reactive responses, developing an approach mode (instead of avoidance to context-specific stressors such as assessments and exams), being able to notice automatic negative thoughts and build a new relationship to one’s thinking, and finally becoming aware of the importance of kindness and self-care when feeling overwhelmed or under the pressure of workload. The underlying approach of MBCT delivery in this context is to support students to meet their vulnerabilities with kindness. It is its “allowed vulnerability” which strengthens coping reserve and improves well-being.

## 6 Discussion

A recent review of ongoing mindfulness training in medical schools only reported on training in the USA, Australia, and Canada, overlooking activity in the UK [34]. This article corrects this oversight by describing mindfulness training in a UK medical school. The most recent review of the effect of mindfulness on health professionals concluded there is a relative paucity of published qualitative examinations of mindfulness interventions with clinicians or trainees [21]. Our research offers the first piece of qualitative work exploring medical student’s experiences of an-eight week training on mindfulness in a UK setting. The findings show that mindfulness training positively influences the way students approach and reflect on their well-being and education within the medical education context. Observations made by other teachers delivering mindfulness in the US put forward the following factors for why mindfulness training has an impact: the effects of group dynamics and the sharing of experiences with others in a similar situation; the didactic content of the course, including learning about how the mind works and how stress impacts one’s life; learning ways of coping with stressors and the importance of self-care; the experiential aspects of the course, including exercises in mindful communication and mind-body connection; the effect of developing a meditation practice; and the impact of role-modelling by instructors [34]. These teacher’s observations are now substantiated by the students’ verbatim data we have explored in our qualitative findings. Another gap in the literature has been the lack of a conceptual model explaining why mindfulness approaches may be particularly helpful amongst medical students. We have developed a theoretical model of the medical student’s “stress signature” [26], relating this to systemic stressors (such as workload and cultures of medicine) shown in Figure 1, in order to map how mindfulness may break a learnt cycle of specific vulnerability and maladaptive coping strategies. Our model presented here has many resonances with research synthesising qualitative work in order to explore processes of therapeutic changes associated with MBCT [35]. For example, research highlights the importance of a shift in feelings towards the self, including an ability to give time for nourishing activities in response to mounting pressures [36]. As Dyrbye and Shanafelt argue, “we must help students to recognise that caring for oneself is an essential part of being a doctor” (p. 344) [37].

Other research interested in students’ well-being describes a “coping reservoir” which can be replenished or drained by various factors, both individually and systemically, leading to either burnout or greater resilience [38]. Mindfulness-based approaches are recommended as one way to “optimize the coping reserve of students” (p. 49) [38], but research has not explored how mindfulness may achieve this for medical students. Our findings have explored, from the students’ perspective, how mindfulness training has facilitated a greater sense of resiliency and coping reserve through the development of awareness. In particular, our findings describe students developing an awareness of judgemental thoughts alongside the cultivation of kinder thoughts about oneself, coupled with a greater sense of flexibility and balance in their approach to work demands. All these factors combine to replenish the coping reserve. Epstein describes self-knowledge achieved through a “curiosity about feelings, thoughts and behaviours without attempting to suppress or label them as good or bad” (p. 837) [18] as the key not just for self-care (and coping reserve) but improved patient care.

Our findings on the impact of mindfulness training upon empathy and communication skills relate to work exploring mindfulness and professionalism [18–20]. Epstein describes “*attentive observation*” (p. 7) [19] of oneself, the patient, and the problem as fundamental to professional and compassionate care. Students in our study struggled to define what they had learnt on the mindfulness course as “traditional communication skills” taught elsewhere in their curriculum. Instead, they described shifts in how they related and communicated with patients brought about through two types of “attentive observation.” First, by becoming more aware of their own thought processes, students in our study describe an ability to “listen more attentively to patients” [18]. Second, by “recognising bias and judgments” [18] in themselves and others, students describe deliberately distancing themselves from such judgments and being able to see the person living with an illness. In both examples, developing inner awareness and self-knowledge was the starting point for professional development in empathy and communication: Our findings are already replicated further upstream in health care professional training. For example, KB, as a collaborator of the Life of Breath project, is currently teaching practical, simple breath-body-mind techniques to clinical professionals to draw on when under stress. KB teaches and researches practices to promote “active” self-care and self-compassion amongst healthcare professionals, which in turn enhances clinical empathy and compassionate communication (https://lifeofbreath.org/event/breath-body-mind-integration/).

### 6.1 Limitations

Our sample size for the qualitative study was small although each interview was long and in-depth. Combined with the free text survey data, we reached saturation in key themes during analysis. At the time of data collection, LR was a final year medical student. Her “social and clinical proximity” [39] to those she interviewed may have been off-putting for some students, and this may be one reason why only six students accepted an invitation to be interviewed. However, based on the deep and honest disclosure of the data, our perspective is that LR’s social and clinical proximity facilitated medical students’ open discussion of their experiences. By inviting a final year medical student to interview other medical students, our work can be aligned with the approach adopted in service-user collaborative research [28] and by feminist scholars who advocate the following: “in most cases, the goal of finding out about people through interviewing is best achieved when the relationship of interviewer and informant is nonhierarchical and when the interviewer is prepared to invest his or her own personal identity in the relationship” (p. 41) [40]. Further research should adopt a longitudinal design, so any benefits of mindfulness training can be explored and assessed as medical students transition from student to junior doctor. More public engagement work is needed to increase awareness amongst medical schools of the value of offering the full 8-week course in mindfulness training.

### 6.2 Implications for Practice

The GMC report “Supporting Medical Students with Mental Health Conditions” (2013) wants medical students to seek help before it becomes a “fitness to practice” concern and wants medical schools to put preventive measures in place to promote good mental health and well-being in their students, including “providing sessions on techniques such as mindfulness and meditation, which some people find useful to help them manage their stress levels” (p. 22) [15]. The findings presented here offer the first qualitative account of medical students’ experiences of an ongoing mindfulness program in the UK, set up to support struggling students and promote students’ well-being. In Australia, a form of mindfulness training is now incorporated into the medical curriculum, whereas in the UK, mindfulness for medical students is still in development with many questions still left unanswered, such as whether mindfulness training should be part of the core curriculum or an optional and self-selected component and whether mindfulness should form one part of a larger well-being program (as in Australia) or be an intensive 8-week course in mindfulness-based cognitive therapy (as explored in this UK example). For example, in New Zealand, students can access resources under one umbrella website known as CALM (http://www.calm.auckland.ac.nz/). This may be a useful prototype for developing and offering online support for medical students, in addition to offering 8-week courses, within a UK setting. A UK-wide survey is now needed to ascertain how other medical schools in the UK are implementing GMC guidelines in this area and how this is compared to what medical schools are delivering in Australia, New Zealand, Canada, and the USA.

## Figures and Tables

**Table 1:** Course content of the 8-week mindfulness training.

Week	Theme	Mindfulness meditation practices and cognitive exercises
1	Automatic pilot	Mindfulness of eating Mindfulness of body sensation
2	Barriers to awareness	Mapping thoughts/feelings/sensations/impulses Mindfulness of daily activity
3	Mindfulness of breath and body	Mindfulness of breathing and movement 3-minute breathing space regular practice Exploring our reactions to the pleasant
4	Staying present	Seeing and hearing meditation Looking at reactivity and maladaptive coping strategies Using the breath as an anchor to awareness Exploring our reactions to the unpleasant
5	Allowing and accepting	Mindfulness of breathing-extended instructions Relating differently to difficulty sea of reactions 3-minute breathing space coping response
6	Thoughts are not facts	Introducing difficulty within a meditation Sea of reactions working with automatic negative thoughts
7	Self-care and action plans	Mindfulness of breath- and body-extended instructions Links between activity and mood nourishing and -depleting activities Identifying a stress signature
8	Using what has been learnt	Review of course using what has been learnt Finalise action plan

**Table 2:** Recruitment to the qualitative study.

Year of study when attending the mindfulness course	Gender	Reasons for attending the course	Who referred them to the mindfulness course
2	F	Shift from academic learning to clinical placements in year 2 was stressful	Student advisor
3	F	Feeling stressed. Wanting to manage thoughts/feelings. Seeing benefit of mindfulness in a friend	Student advisor
1	F	Feeling stressed, isolated, withdrawn	Student advisor
2	F	Feeling down, subdued, and actively stressed	Student advisor
2	M	Finding the course stressful. Wanting to learn coping mechanisms.	Student advisor
3	F	Recovery from eating disorder and learning to cope better with stress	Student GP

**Table 3:** Topic guide for qualitative interviews.

Reasons for joining the course
Self-care and coping strategies before the course
Attitudes to help-seeking
Previous experience of barriers to help-seeking
Stigma surrounding help-seeking
Challenges of the course
Managing the commitment of the course
Experience of group context
Experience of mindfulness practices and cognitive exercises
What has been learnt
Impacts and changes
Ongoing practice
Course content and structure
Implementation within curriculum

**Table 4:** Open-ended survey questions for free text responses.

Why did you come on this course and why did you stay?
What if anything have you learned that has been useful?
What changes, if any, have you noticed?
If you plan to continue to use mindfulness, what will you use in daily life?
What were your biggest obstacles?
On a scale of 1 (not at all important) to 10 (extremely important), how important has this course been to you? Please say why you have given it this rating
What was it like learning mindfulness in a small group?
What have you found least helpful about the course?
Any other comments?

**Table 5:** Year of study of 57 survey responders who attended an 8-week course on mindfulness between 2011 and 2015.

Year of study	Number of students, *N* = 57
1	8
2	15
3	17
4	7
5	3
Unknown/not recorded	4
